# Identification of the Biocontrol Effect of *Bacillus velezensis* LYH8 Against *Fusarium* Head Blight of Wheat

**DOI:** 10.3390/jof12030199

**Published:** 2026-03-10

**Authors:** Yihua Liao, Jiayi Shen, Tian Yang, Huijuan Peng, Tingwei Qi, Yan Li, Chengcheng Li

**Affiliations:** Key Laboratory of Green and Efficient Production of Crops in the Middle Reaches of the Yangtze River (Co-Constructed by the Ministry and the Province), Ministry of Agriculture and Rural Affairs, College of Agriculture, Yangtze University, Jingzhou 434025, China; liaoyihua0815@163.com (Y.L.); 2023750192@yangtzeu.edu.cn (J.S.); 2022004773@yangtzeu.edu.cn (T.Y.); 2024720966@yangtzeu.edu.cn (H.P.); 2023005145@yangtzeu.edu.cn (T.Q.)

**Keywords:** *Fusarium graminearum*, *Bacillus velezensis LYH8*, antifungal activity, deoxynivalenol, *Fusarium* head blight management

## Abstract

*Fusarium* head blight (FHB), caused by *Fusarium graminearum*, is a fungal disease that severely affects wheat. The mycotoxins it produces, such as deoxynivalenol (DON), pose serious risks to human and animal health. In this study, a biocontrol strain, LYH8, was isolated from local sources in Jingzhou, Hubei Province. Plate confrontation assays demonstrated that LYH8 effectively inhibited the mycelial growth of *F. graminearum*, with an inhibition rate of 43%, and induced morphological abnormalities such as hyphal swelling and shrinkage. Based on *16S rRNA* and *gyrB* gene sequencing and phylogenetic analysis, LYH8 was identified as *Bacillus velezensis*. In vivo experiments showed that disease severity in wheat coleoptiles and spikes was significantly reduced by treatment with LYH8 by 75–85%, and the accumulation of DON and its deoxynivalenol-3-glucoside (D3G) in grains was decreased by 20–22%. Further transcriptome analysis revealed that it affects pathogen growth by regulating amino acid biosynthesis, ribosomal biosynthesis, carbon metabolism pathways, and the catalytic activities of related genes. In summary, LYH8 significantly controlled FHB through multiple mechanisms, including inhibiting mycelial growth, reducing infection, and blocking toxin synthesis, demonstrating strong biocontrol potential.

## 1. Introduction

Wheat is a core global food crop, and its safe production is crucial for overall food security [1]. Driven by global climate change and the evolution of farming systems, the prevalence of *Fusarium* head blight (FHB) continues to expand, making it a critical factor threatening wheat yield and food safety [2]. The disease not only leads to substantial yield losses but also poses a significant threat to food safety and human and animal health due to the mycotoxins it produces, such as deoxynivalenol (DON). Consequently, the control of these contaminants has received considerable attention and has been established as a key international food safety indicator [3,4]. In major wheat-growing regions of China, such as the Huang-Huai area of Hubei Province, the incidence and severity of FHB have increased significantly, influenced by changing precipitation patterns and widespread adoption of straw incorporation [5]. Current control measures rely primarily on a limited number of disease-resistant cultivars and chemical fungicides. However, these approaches face challenges including pathogen adaptation, increasing fungicide resistance, and scarcity of resistant germplasm resources. The long-term reliance on fungicides with a single mode of action, such as carbendazim, has contributed to environmental contamination and ecological imbalance, accelerating the development of pathogen resistance and exacerbating disease spread [6]. In recent times, biological control has offered a novel approach for managing FHB in wheat due to its high efficiency, safety, and environmental friendliness. Among various biocontrol microorganisms, the genus *Bacillus* has become one of the most extensively studied and applied groups, owing to its strong stress tolerance, broad-spectrum antimicrobial activity, and diverse bioactive metabolites. For instance, strains such as *Bacillus amyloliquefaciens* and *Bacillus subtilis* have demonstrated promising potential in controlling fungal diseases [7,8]. With advances in taxonomy and genomics, increasing attention has been focused on a group of strains closely related to *B. amyloliquefaciens* that exhibit outstanding biocontrol capabilities such as *Bacillus velezensis.*

*Bacillus velezensis* is a Gram-positive plant growth-promoting rhizobacterium (PGPR) with demonstrated potential for biological control [9]. It primarily functions through a dual mechanism involving direct inhibition and induced systemic resistance. The bacterium is capable of synthesizing and secreting a variety of metabolites with broad-spectrum antimicrobial activity, which act directly against phytopathogens [10]. These primarily include lipopeptide antibiotics such as surfactin, iturin, and fengycin, which disrupt the integrity of the fungal cell membrane [11]. Additionally, it can produce cell wall-degrading enzymes such as chitinase and glucanase, as well as volatile antimicrobial compounds, thereby achieving effective suppression during the early stages of pathogen infection [12]. Furthermore, *B. velezensis* enhances overall plant disease resistance and growth vigor through multiple indirect pathways. It can secrete phytohormones, such as auxins and cytokinins, to promote root development; synthesize siderophores to improve iron competition; and rapidly colonize the rhizosphere to occupy ecological niches, thereby reducing the survival space for pathogens [13]. These two mechanisms work synergistically, effectively controlling pathogen spread in the short term while enhancing the host plant’s long-term health and stress tolerance. This synergy underscores its systemic value as a multifunctional biocontrol agent in sustainable agriculture. Due to its broad spectrum antimicrobial activity, *B. velezensis* has become a mainstream biocontrol strain both domestically and internationally, and is widely applied in agricultural green management [14].

As a biocontrol agent, *B. velezensis* has shown promising application potential in agriculture and animal husbandry. Specifically, strain ZF516 shows effective biocontrol activity against tomato bacterial canker [15]. Foliar application of *B. velezensis* increased sweet potato yield, enhanced root antioxidant enzyme activity, and induced ultrastructural changes in leaf chloroplasts, resulting in denser stromal architecture and enlarged intracellular starch granules [16]. Although *B*. *velezensis* has demonstrated potential in controlling various crop diseases, studies on its inhibitory effects against *Fusarium graminearum* growth and DON synthesis remain limited, particularly regarding the integration of transcriptomic analysis to elucidate its mechanisms of action.

This study systematically investigated a newly isolated *Bacillus* strain LYH8 from Jingzhou. Phylogenetic analysis based on multiple genes and physiological/biochemical characterization confirmed its taxonomic identity. Building on this, we further examined morphological changes in *F*. *graminearum* hyphae after co-culture with this strain, evaluated its in vitro antagonistic activity, and assessed its effect on the biosynthesis of the key virulence factor DON and its derivative deoxynivalenol-3-glucoside (D3G). Concurrently, transcriptomic analysis was performed to preliminarily elucidate its potential mode of action. This study aimed to explore the biocontrol potential of strain LYH8, a preliminary exploration of the research gap in the control of *F. graminearum* and inhibition of its toxin production by *B. velezensis*, providing a theoretical and technical foundation for its future development as a green biocontrol agent against FHB in wheat.

## 2. Materials and Methods

### 2.1. Tested Samples and Culture Media

The wild-type *F. graminearum* PH-1 and *B. velezensis* LYH8 were both preserved in the Molecular Biology Laboratory of the College of Agriculture, Yangtze University. The culture media used were as follows: Potato dextrose agar (PDA) medium; LB solid medium; LB broth; mung bean broth medium and YEPD medium (All media were prepared in-house by the laboratory, College of Agriculture, Yangtze University, Jingzhou, China).

### 2.2. Identification of Strain LYH8

Molecular identification of the strain was performed by amplifying and sequencing the *16S rRNA* gene (using primers 27F/1492R) and the *gyrB* gene (using primers UP1f/UP2r) (Supplementary Table S1) [17]. After amplification verification by gel electrophoresis, the PCR products were sent to Sangon Biotech (Shanghai) Co., Ltd. (Shanghai, China) for sequencing. Homology analysis was performed using the BLASTn program on NCBI. A phylogenetic tree was constructed with the maximum likelihood method in MEGA 11.0 software. The tree included eight reference strains representing closely related species within the genus *Bacillus: B. velezensis* strain 8-4, *B. amyloliquefaciens* strain BCRC 17,038, *Bacillus atrophaeus* strains LSSC3, BCRC 17,123, and BCRC 17,530, *Bacillus licheniformis* strain ZW3, *Bacillus sonorensis* strain BCRC 17,532, and *Bacillus cereus* ATCC 14,579. *B. cereus* ATCC 14,579 was used as an outgroup. The reliability of the tree topology was assessed with 1000 bootstrap replicates.

### 2.3. In Vitro Antagonism Assay

The antagonistic activity of strain LYH8 against *F. graminearum* PH-1 was assessed using a dual-culture plate assay: A mycelial plug (6 mm in diameter) of *F. graminearum*, precultured at 25 °C for 3 days, was placed at the center of a PDA plate. Four small wells were made in a cross pattern at a distance of 2.5 cm from the center using a 6 mm diameter cork borer. Each well was filled with 40 µL of LYH8 bacterial suspension (OD_600_ = 0.6, 1 × 10^8^ CFU·mL^−1^), which had been preincubated at 37 °C for 6 h. In the control group, an equal volume of sterile medium was added to the corresponding wells. All treatments were performed with three biological replicates. After incubation at 25 °C for 3 days, the colony diameter from the central *F. graminearum* hyphal plug was measured, and the inhibition rate was calculated according to the following formula:

$$Inhibition\;rate\;(\%)=\frac{Control\;colony\;diameter\;-\;Treatment\;colony\;diameter}{Control\;colony\;diameter}\;\times \;100$$

### 2.4. Effect of the Cell-Free Fermentation Filtrate of LYH8 on the Mycelial Growth of F. graminearum

To prepare the cell-free fermentation filtrate of the strain, a single colony of LYH8 was inoculated into 50 mL of LB broth and incubated at 37 °C with shaking at 200 r·min^−1^ for 24 h to obtain the primary culture, which was adjusted to an OD_600_ of 0.8. One milliliter of this culture was then transferred into 100 mL of fresh LB broth and further incubated with shaking for 4 days. The fermentation broth was centrifuged to collect the supernatant, which was then sterilized through a 0.22 μm filter membrane to obtain sterile fermentation broth and stored at 4 °C for subsequent use. The sterile fermentation broth was incorporated into PDA medium at the concentrations of 1%, 5%, 10% and 20%, respectively, followed by plate pouring. After solidification, a pathogen hyphal plugs was placed at the center of each plate. PDA plates without fermentation broth served as the control, with three replicates set for each concentration. After incubation at 25 °C in the dark for 3 d, the colony diameters were measured.

### 2.5. Mycelial Structure of F. graminearum and LYH8 Under Co-Cultivation Conditions

To investigate the effects of strain LYH8 on the mycelial growth of *F. graminearum* at different time points, mycelial discs of *F. graminearum* were inoculated into mung bean broth medium and cultured with shaking at 25 °C and 200 r·min^−1^ for 3 d. The spore suspension was collected by filtration and adjusted to a concentration of 1 × 10^6^ CFU·mL^−1^. Equal volumes of LYH8 bacterial suspension and the pathogenic fungal spore suspension were co-inoculated into YEPD medium, with the treatment without LYH8 as the control. The cultures were incubated with shaking at 25 °C and 200 r·min^−1^ for 24 h and 48 h, respectively. At each time point, 1 mL of fungal samples was collected, and their morphology was observed under a light microscope (Nikon Corporation, Tokyo, Japan).

Furthermore, the ultrastructure of hyphae was observed using a scanning electron microscope (VEGA3, Tescan, Czech Republic) [18]. Aerial hyphae at the edge of the inhibition zone were scraped off, and an appropriate amount of mycelial blocks were excised with the underlying medium removed. The samples were fixed with 2.5% glutaraldehyde (Aladdin Biochemical Technology Co., Ltd., Shanghai, China) for 20 min and rinsed with double distilled water, followed by gradient dehydration in 50%, 70%, 80%, 90% and 100% ethanol (Fudon Biochemical Technology Co., Ltd., Hubei, China)for 20 min each. Subsequently, the samples were treated with 75% and 100% tert-butanol (Aladdin Biochemical Technology Co., Ltd., Shanghai, China), frozen for 10 min, and vacuum-dried for 48 h. After gold sputtering, the ultrastructures were observed under the microscope. The specific imaging parameters were as follows: voltage of 20.0 kV, working distance of 2.5–3.0 mm, magnification range of 2.5 kx, and detector type of SE.

### 2.6. Control Efficacy of LYH8 Against F. graminearum

Seeds of wheat cultivar R39 were surface-sterilized with 2% sodium hypochlorite solution (Aladdin Biochemical Technology Co., Ltd., Shanghai, China) for 15 min, thoroughly rinsed, and then placed on moist filter paper for germination. After radicle emergence, the seeds were transplanted into seedling trays for cultivation. When the coleoptile length of seedlings reached 2–3 cm, 10 μL of LYH8 bacterial suspension (OD_600_ = 0.6, 1 × 10^8^ CFU·mL^−1^) was precisely inoculated onto the coleoptile. After 24 h, the same inoculum volume of fungal suspension was applied to the same site, and the lesion length was measured on the seventh day post-inoculation.

In the wheat spike inoculation assay, the spikes were sprayed with the LYH8 bacterial suspension at the aforementioned concentration at the anthesis stage. After 24 h, the 5th spikelet from the base of each spike was selected, and 10 µL of the spore suspension was injected into it. The inoculated spikes were spray-moisturized and bagged and incubated at 25 °C for 2 d. At 14 d post-inoculation, the disease incidence was recorded, and diseased grains were collected.

### 2.7. Determination of Toxin Content in Wheat Grains

Diseased grain samples were ground in liquid nitrogen, and an acetonitrile-water mixture (84:16, *v*/*v*) (Aladdin Biochemical Technology Co., Ltd., Shanghai, China) was used as the extraction solvent. The mixture was vortexed for 30 min, sonicated for 1 min, and vortexed again for 30 min. Subsequently, the extract was purified by solid-phase extraction column, and 800 µL of the eluate was collected and vacuum-dried. The obtained residue was derivatized with 50 μL of TMS silylation reagent (TMSI: TMCS = 100:1, *v*/*v*), and the tube was vortexed for 10 min. Subsequently, 500 μL of isooctane and 500 μL of water were added and mixed. The supernatant was collected, filtered, and analyzed by gas chromatography-mass spectrometry (GC-MS) using a TG-5MS capillary column (60 m × 0.25 mm i.d. × 0.5 μm film thickness). Helium was used as the carrier gas at a constant flow rate of 1.5 mL/min. The column temperature program was set as follows: initial temperature 140 °C held for 0.5 min, then increased at a programmed rate to 300 °C and held for 20 min. Ionization was performed in electron impact (EI) mode at 70 eV. The content of deoxynivalenol (DON) was calculated according to the following formula: C = (C_0_ ×1.8)/m, where C denotes the DON concentration in µg/g, C_0_ the concentration measured by GC/MS, and m the sample weight in grams.

### 2.8. Gene Expression of F. graminearum

Mycelial discs of *F. graminearum* were inoculated into mung bean broth medium and cultured with shaking at 25 °C and 200 r·min^−1^ for 3 d to prepare a spore suspension (1 × 10^6^ CFU·mL^−1^). The spore suspension was co-cultured with the LYH8 bacterial suspension (OD_600_ = 0.6, 1 × 10^8^ CFU·mL^−1^) in liquid YEPD medium, followed by continuous shaking cultivation at the aforementioned temperature and rotation speed for 48 h. Total RNA of the samples was extracted using the RNAprep Pure Plant Kit (Tiangen Biotech Co., Ltd., Beijing, China). RNA integrity was detected via the RNA Nano 6000 Assay Kit coupled with the Agilent Bioanalyzer 2100 system (Agilent Technologies, Inc., Santa Clara, CA, USA). RNA samples of sufficient quality were sent to Wuhan Yuehang Biotechnology Co., Ltd., Wuhan, China, for sequencing on the Illumina NovaSeq 6000 platform [19,20].

The raw sequencing data in FASTQ format were preprocessed with an in house Perl script to obtain high quality clean reads, which were subsequently aligned using Hisat2 2.2.1 software. Based on the alignment outcomes, differentially expressed genes (DEGs) were identified via DESeq2 with the following screening criteria: |log_2_ (fold change) | ≥ 2, *p* ≤ 0.01, and false discovery rate (FDR) < 0.01 [21]. Functional annotation and enrichment analysis of the DEGs were further conducted using the Gene Ontology (GO: http://www.geneontology.org, accessed on 16 September 2025) and Kyoto Encyclopedia of Genes and Genomes (KEGG: https://www.genome.jp/kegg, accessed on 16 September 2025) databases. To verify the reliability of the sequencing results, up- and down-regulated DEGs were separately selected to design gene-specific primers, and validation was performed via the 2^−ΔΔCT^ method with the qRT-PCR protocol. (Supplementary Table S1).

### 2.9. Statistical Analysis

Statistical analysis was performed using SPSS Statistics 20.0 software (IBM Corp., Armonk, NY, USA). An unpaired Student’s *t*-test was used for comparisons between two groups, and one-way analysis of variance (ANOVA) followed by Tukey’s multiple comparison test was employed for multiple group comparisons. A value of *p* < 0.05 was considered statistically significant [22]. The height of the bars in the bar charts corresponds to the mean value of biological replicates, while the error bars represent the standard error of the mean.

## 3. Results

### 3.1. Results of Strain LYH8 Identification

**Figure 1:**
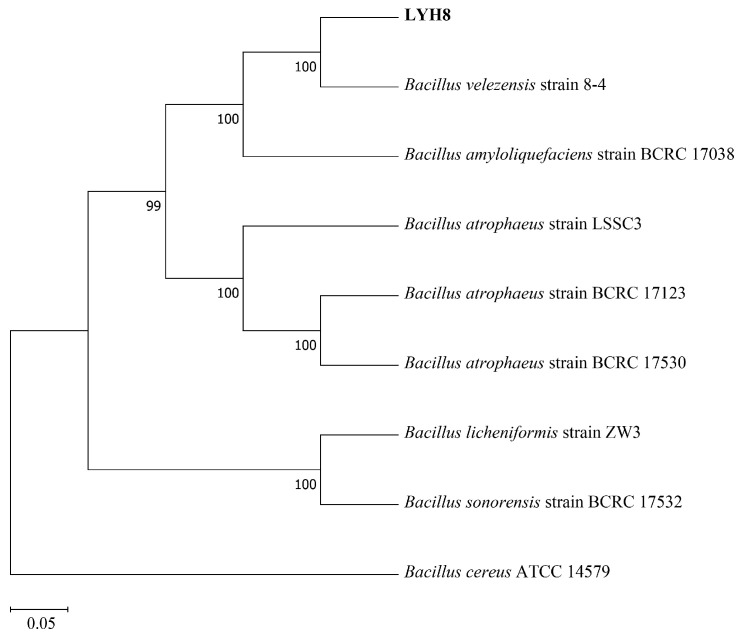
Phylogenetic tree of strain LYH8. Phylogenetic tree of strain LYH8 constructed by the maximum likelihood method based on concatenated sequences of the *16S rRNA* and *gyrB* genes. Numbers at the branch nodes indicate bootstrap support values based on 1000 replicates; the scale bar at the bottom represents genetic distance.

Based on molecular phylogenetic analysis, strain LYH8 was identified as *B*. *velezensis*. BLAST alignment of its *16S rRNA* and *gyrB* gene sequences showed that LYH8 shared 99.96% (2526/2527 bp) sequence identity with *B. velezensis* strain 8-4, and 99.20% (2507/2527 bp) identity with *B. amyloliquefaciens* strain BCRC 17,038. Phylogenetic tree construction using closely related *Bacillus* strains further revealed that LYH8 clustered with *B. velezensis* strain 8-4 in a well-supported clade, with a bootstrap support value of 100% (Figure 1).

### 3.2. Antifungal Activity of Strain LYH8 Against F. graminearum

**Figure 2:**
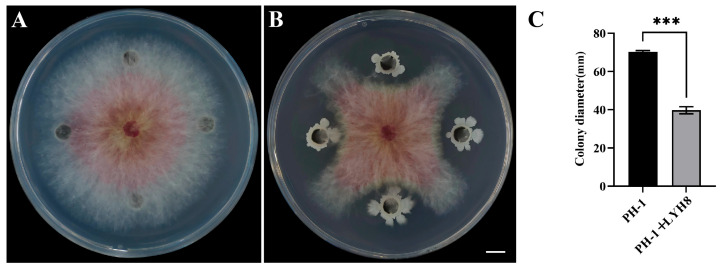
Antifungal activity of strain LYH8 against *F. graminearum* PH-1. (**A**) *F. graminearum* (**B**) *F. graminearum* PH-1 + LYH8. (**C**) Colony diameters of the two treatments. All experiments were performed in triplicate on different days, and consistent results were obtained. Statistical significance was determined by unpaired Student’s *t*-test (*** *p* < 0.001 vs. PH-1).

Strain LYH8 exhibited strong antifungal activity against the tested *F. graminearum* strain PH-1. The results of the dual culture assay showed that the inhibition rate of this strain on the mycelial growth of PH-1 reached as high as 43% (Figure 2).

### 3.3. Effects of Cell-Free Fermentation Broth on Mycelial Growth of F. graminearum

**Figure 3:**
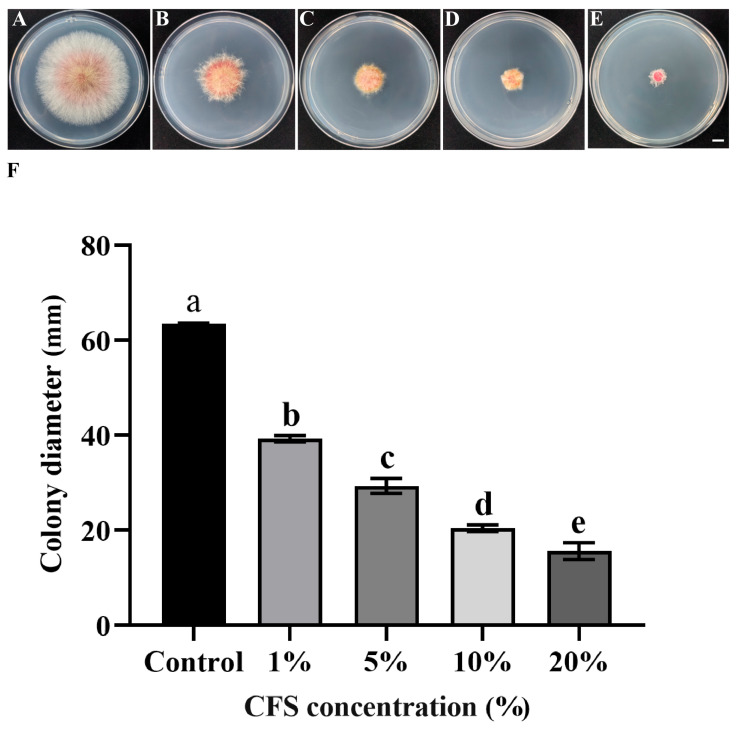
In vitro antifungal effect of strain LYH8 against *F. graminearum* treated with its cell-free fermentation supernatant at different concentrations. (**A**–**E**) Mycelial growth morphology of *F. graminearum* PH-1 on PDA plates after treatment with the supernatant at various concentrations (**F**) Colony diameters (mm) of *F. graminearum* PH-1 treated with the supernatant at different concentrations. Scale bar: 1 cm. All experiments were performed in triplicate on different days, and consistent results were obtained. Statistical analysis was conducted using Tukey’s Honestly Significant Difference (HSD) test; different letters above the bars indicate significant differences between groups (*p* < 0.05).

Based on the phenotypic characteristics and colony diameter measurements, the cell-free fermentation broth of strain LYH8 exhibited a significant inhibitory effect on the mycelial growth of *F. graminearum* PH-1 (Figure 3A–F). Its antimicrobial activity was positively correlated with the increase in broth concentration gradient.

### 3.4. Effects of LYH8 on Mycelial Morphology of F. graminearum

**Figure 4:**
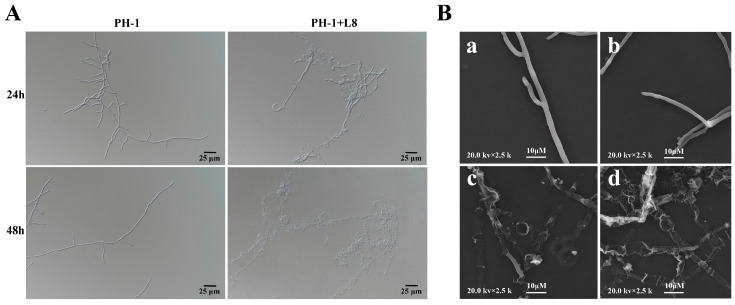
Effect of LYH8 treatment on the hyphal morphology of *F. graminearum*. (**A**) Observation results of the control and treatment groups under light microscopy at 24 h and 48 h. (**B**) Observation results of hyphae from the edge of the inhibition zone after 3 days of culture using scanning electron microscopy. (**a**,**b**): *F. graminearum* PH-1; (**c**,**d**): *F. graminearum* PH-1 + LYH8.

Comparative observations via light microscopy and scanning electron microscopy revealed significant morphological differences in the hyphae of *F. graminearum* between the untreated control group and the LYH8-treated group. The hyphae in the control group displayed a smooth surface, uniform diameter, and regular arrangement. In contrast, in the treatment group, partial hyphae exhibited fragmentation with obvious terminal swelling and bulging, accompanied by abnormal morphological features such as structural collapse and shrinkage (Figure 4). These results indicated that LYH8 significantly inhibited the hyphal growth of *F. graminearum*.

### 3.5. Control Efficacy of Strain LYH8 Against Wheat FHB

**Figure 5:**
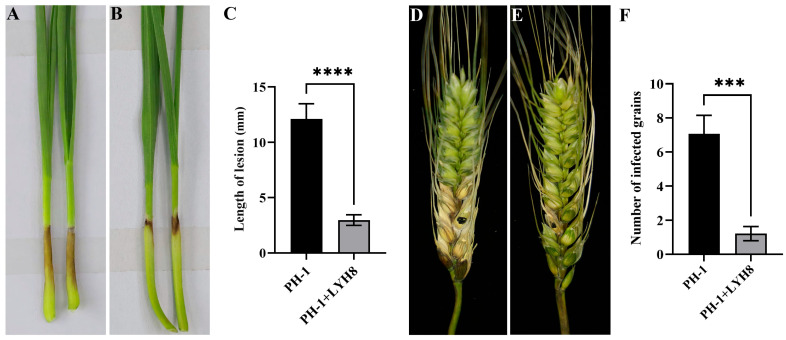
Control effect of strain LYH8 against FHB in wheat. (**A**) Wheat coleoptiles infected with *F. graminearum* PH-1 for 7 days. (**B**) Wheat coleoptiles infected with *F. graminearum* PH-1 + LYH8 for 7 days. (**C**) Statistical analysis of lesion length on wheat coleoptiles after infection. (**D**) Wheat spikes infected with *F. graminearum* PH-1 for 14 days; (**E**) Wheat spikes infected with *F. graminearum* PH-1 + LYH8 for 14 days; (**F**) Statistical analysis of the number of diseased grains on wheat spikes after infection. All experiments were independently repeated three times on different days, and consistent results were obtained. Statistical significance was determined by unpaired Student’s *t*-test (**** *p* < 0.0001, *** *p* < 0.001 vs. PH-1).

The effect of strain LYH8 on the pathogenicity of *F. graminearum* was evaluated via wheat coleoptile and single spike inoculation assays. The results showed that the disease severity on coleoptiles and wheat spikes was significantly reduced after LYH8 treatment (Figure 5). Specifically, the lesion length at the coleoptile base in the control group was approximately 12 mm, with the number of diseased grains per spike reaching 7. In contrast, in the LYH8-treated group, the lesion length at the coleoptile base was only about 3 mm, and the number of diseased grains per spike was reduced to 1.

### 3.6. Effects of LYH8 onDON Toxin Biosynthesis

**Figure 6:**
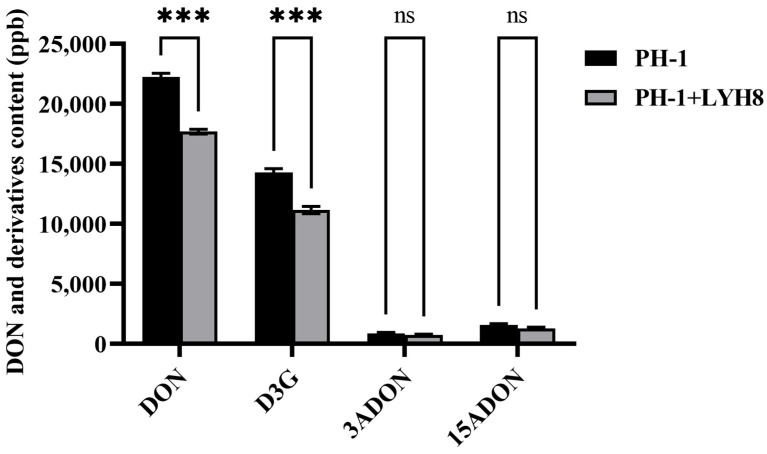
Statistical analysis of DON and its derivative D3G content in infected wheat grains. All experiments were independently repeated three times on different days, and consistent results were obtained. Statistical significance between PH-1 and PH-1 + LYH8 was determined by unpaired Student’s *t*-test (*** *p* < 0.001; ns, not significant).

To investigate whether LYH8 affects the biosynthesis of DON and its derivative D3G in *F. graminearum* PH-1, toxin contents in infected wheat grains from treated and untreated groups were compared. The results showed that LYH8 treatment significantly inhibited the accumulation of DON and D3G in grains, with no effect on 3-acetyldeoxynivalenol (3ADON) and 15-acetyldeoxynivalenol (15ADON) (Figure 6). LYH8 effectively inhibited DON biosynthesis and modulated the mycotoxin synthesis pathway.

### 3.7. Transcriptomic and Functional Enrichment Analyses of F. graminearum in Response to LYH8 Treatment

**Figure 7:**
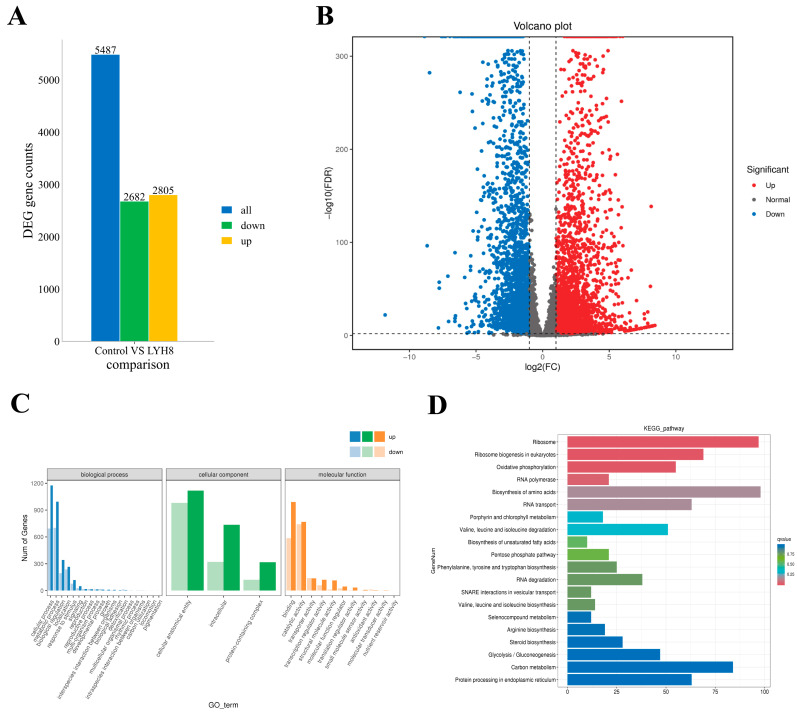
Changes in gene expression of *F. graminearum* PH-1 after treatment with strain LYH8. (**A**) Numbers of up- and down-regulated DEGs among all identified DEGs (**B**) Volcano plot of DEGs between the control and LYH8 treatment groups. Red dots represent up-regulated DEGs, blue dots represent downregulated DEGs, and gray dots indicate non-DEGs. (**C**) Histogram of Gene Ontology (GO) functional classification. DEGs were classified into three categories: biological process, cellular component, and molecular function. (**D**) Histogram of the top 20 KEGG pathways ranked by the smallest q-values.

Analysis of DEGs between the control and LYH8-treated groups identified 5847 DEGs with the screening criteria of |log_2_FC| ≥ 2 and FDR ≤ 0.01, including 2682 downregulated and 2805 upregulated genes (Figure 7A,B).

GO analysis revealed significant enrichment of genes related to metabolic/cellular processes and binding/catalytic activity, indicating that LYH8 broadly disrupts fundamental physiological activities (Figure 7C). KEGG enrichment analysis (Figure 7D) identified key pathways perturbed by LYH8, mechanistically explaining the observed inhibition of hyphal growth and virulence. The analysis revealed a coordinated transcriptional response involving upregulation of stress-related pathways and downregulation of core metabolic processes:

Upregulated genes: Steroid biosynthesis (*FGSG_05740, FGSG_04092*) under LYH8-induced stress, the fungus enhances ergosterol synthesis to maintain membrane integrity; peroxisome function (*FGSG_05011*) increased expression indicates activation of detoxification mechanisms to counteract oxidative stress; and ribosome biosynthesis (*FGSG_06724*) reflecting the fungus’s attempt to maintain protein synthesis capacity under adverse conditions. Downregulated genes: The key glycolytic gene *FGSG_07803* (glycolysis/gluconeogenesis) limits ATP supply for hyphal elongation; suppression of *FGSG_04903* and *FGSG_12603* (amino sugar and nucleotide sugar metabolism) impairs cell wall biosynthesis; and downregulation of nitrogen metabolism (*FGSG_10453*) limits nitrogen utilization for amino acid and nucleotide synthesis, indirectly affecting growth and toxin production. DON biosynthesis: *TRI* genes (*TRI6, TRI10*) were significantly downregulated, providing direct transcriptional evidence for reduced DON production. qRT-PCR validated the downregulation of these *TRI* genes, confirming that LYH8 inhibited mycotoxin synthesis at the transcriptional level.

LYH8 inhibited *F. graminearum* through a dual mechanism: upregulating stress response pathways (steroid biosynthesis, peroxisome function, ribosome biosynthesis) to cope with induced stress while downregulating core metabolic processes (glycolysis, amino sugar metabolism, nitrogen metabolism) to limit energy supply and growth-related biosynthesis. Combined with the direct suppression of *TRI* genes, these transcriptional changes provide a molecular basis for the observed inhibition of both hyphal growth and toxin synthesis.

### 3.8. Validation of Transcriptome Data by qRT-PCR

**Figure 8:**
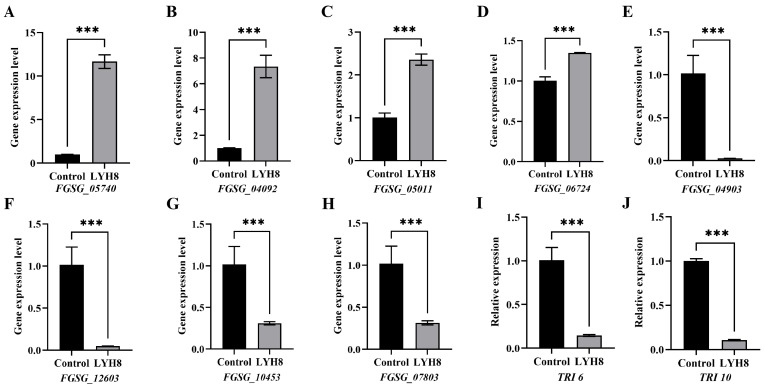
RT-qPCR validation of DEGs identified by transcriptome analysis. (**A**–**D**) Up-regulated DEGs; (**E**–**H**) Downregulated DEGs. (**I**,**J**) Downregulated virulence genes. Note: Asterisks denote highly significant differences at *p* < 0.001 (***).

Based on transcriptomic data, DEGs were screened using the absolute value of log_2_FC, and four upregulated and four downregulated genes were selected for validation. Furthermore, to further verify the inhibitory effect of LYH8 on DON biosynthesis, the key toxin synthesis regulatory genes *TRI6* and *TRI10* were also subjected to quantitative analysis, both of which were downregulated. The results showed a high consistency between RNA-seq and qRT-PCR data, thus confirming the reliability of the transcriptome analysis (Figure 8).

## 4. Discussion

Given the problems of environmental pollution and increased pathogen resistance associated with the chemical control of wheat FHB, eco-friendly biocontrol strategies that are less likely to induce resistance have attracted great attention and become an important development direction in the integrated management system [23]. As a beneficial microbe with broad-spectrum antifungal activity and plant safety, *B. velezensis* holds great value in the field of green biological control [24,25,26]. In this study, a strain of *B. velezensis* LYH8 was used, and it was found to exert significant in vitro and in vivo antagonistic effects against *F. graminearum*. In addition to directly inhibiting the pathogen, *Bacillus* species have also been shown to induce systemic resistance in plants, enhancing the defense capacity of floral tissues against *Fusarium* [27]. This dual mode of action likely contributes to the observed reduction in disease severity on wheat spikes. Relevant studies have shown that *B. velezensis* can inhibit the spore germination of *F. graminearum* by up to 81.67% and reduce the DON content by 66.6% [28].

Crops infected by *Fusarium* can produce various mycotoxins, mainly DON and D3G; 3ADON and 15ADON are acetylated derivatives directly synthesized by the fungus, which are easily hydrolyzed to DON in vivo. DON is the most harmful and prevalent mycotoxin in food crops. Relevant literature indicates that *B. velezensis* SX1302 exhibits strong inhibitory effects against *F. graminearum*, and its cell suspension significantly suppresses DON production with an inhibition rate of 86.89% [29]. The intracellular soluble fraction of a *Bacillus firmus* strain exhibited a detoxification rate of 72.70% [30]. Strain 6W1 could produce volatile organic compounds (VOCs) with antifungal and toxigenic inhibitory activities; this microbial resource holds great potential for development as a biocontrol product, and its VOCs also have high research and application value in plant disease control [31]. Eight antagonistic *Bacillus* strains were isolated from wheat plants, seven of which were *B*. *subtilis*; strain B08, with the strongest antifungal activity, achieved a control efficacy of over 65% against *F. graminearum* [32]. Importantly, LYH8 significantly reduced the accumulation of DON and D3G. As representative mycotoxin virulence factors, DON can promote fungal colonization and suppress host defense responses by inducing tissue necrosis [33]. Therefore, the observed inhibition of toxin synthesis directly contributes to the reduction in fungal pathogenicity. Thus, the toxin-control function of LYH8 is not merely a detoxification process but rather a key mechanism limiting disease development in wheat. Through multi-pathway regulation, it achieved efficient biocontrol, providing a promising microbial resource for the green management of FHB and the mitigation of mycotoxin contamination.

Transcriptomic analysis revealed that LYH8 disrupts the pathogenic system of *F*. *graminearum*. The downregulation of glycolysis and carbon metabolism limits ATP supply, directly constraining the energy-demanding process of polarized hyphal elongation. Concurrent suppression of amino sugar and nucleotide sugar metabolism impairs cell wall integrity, further weakening hyphal morphogenesis and host penetration capability. Meanwhile, the upregulation of steroid biosynthesis and peroxisome function reflects the fungus’s ability to maintain membrane integrity and counteract oxidative damage. Most critically, the significant downregulation of *TRI* genes directly attenuates DON biosynthesis, depriving the fungus of a key virulence factor that suppresses host defenses and promotes tissue colonization. The contradiction between the hyperactive demand for biosynthesis and insufficient energy supply ultimately trapped the pathogen in a metabolic state of dual depletion of energy and resources, effectively inhibiting its hyphal growth and pathogenicity [34]. *Bacillus* strains are widely applied. Under the antagonism of *B. velezensis* F85, the transcriptomic characteristics of *Colletotrichum nicotianae* hyphae changed significantly, and F85 mainly affected hyphal peroxisome regulation and energy generation processes [35]. The molecular mechanism underlying the antifungal activity of secondary metabolites (SMs) from *B. subtilis* J-15 against *Saccharomyces cerevisiae* provides an experimental basis for elucidating the antifungal mechanism of SMs and a theoretical foundation for their application in the control of crop fungal diseases [36].

In summary, *B*. *velezensis* LYH8 protects wheat spikes from *F*. *graminearum* infection through a multi layered mechanism. First, it exerts direct antagonistic effects, causing hyphal morphological damage and reducing fungal biomass. Second, it suppresses *TRI* gene expression at the transcriptional level, limiting DON biosynthesis and thereby attenuating the pathogen’s ability to overcome host defenses. Third, it disrupts core metabolic pathways essential for polarized growth and cell wall integrity while simultaneously triggering responses that deplete cellular resources. This integrated model positions LYH8 as a promising biocontrol agent that not only directly inhibits the pathogen but also reduces its virulence and metabolic capacity, offering a sustainable strategy for the management of wheat FHB. Currently, 32 biopesticide products have been registered, accounting for 5.10% of the total registered pesticides with 11 active ingredients. The main microbial-derived ones include *B. subtilis*, *B. velezensis* C17,271 and *Paenibacillus polymyxa* KN-03, all characterized by high application efficiency and low residue. Notably, pesticides with novel modes of action will become a key research area for wheat FHB control in the future and an effective approach to addressing the resistance issue.

## Data Availability

The raw sequence data reported in this paper have been deposited in the Genome Sequence Archive (Genomics, Proteomics & Bioinformatics 2025) at the National Genomics Data Center (Nucleic Acids Res 2025), China National Center for Bioinformation / Beijing Institute of Genomics, Chinese Academy of Sciences, and are publicly accessible under the accession number CRA038132 at https://ngdc.cncb.ac.cn/gsa (accessed on 9 February 2026).

## Supplementary Materials

The following supporting information can be downloaded at: https://www.mdpi.com/article/10.3390/jof12030199/s1, Table S1: Primers used in the study. The raw sequence data reported in this paper have been deposited in the Genome Sequence Archive (Genomics, Proteomics & Bioinformatics 2025) in National Genomics Data Center (Nucleic Acids Res 2025), China National Center for Bioinformation/Beijing Institute of Genomics, Chinese Academy of Sciences (GSA: CRA038132) that are publicly accessible at https://ngdc.cncb.ac.cn/gsa (accessed on 9 February 2026).
