# Supplementary material for: Identification of the Biocontrol Effect of Bacillus velezensis LYH8 Against Fusarium Head Blight of Wheat
Source: J Fungi (Basel). 2026 Mar 10;12(3):199. doi: 10.3390/jof12030199 (PMC13028092; doi:10.3390/jof12030199)
Supplement: Supplementary file 1 [file jof-12-00199-s001.zip › jof-4170857-supplementary.pdf]

Supporting information

Table S1 Primers used in the study

Primer	Sequence (5'-3')	Application	Source
27F	AGAGTTTGATCMTGGCTCAG	Amplification of *16S rRNA* (NR_116240.1)	https://doi.org/10.3389/fpls.2024.1370440
1492R	GGTTACCTTGTTACGACT		
UP1f	GAAGTCATCATGACCGTTCTGCA	Amplification of *gyrB* (DQ903176.1)	
UP2r	AGCAGGGTACGGATGTGC		
FGSG_05740-F	ACGGATGGTCCCAGAGTTTT	Quantitative real-time PCR of *FGSG_05740* expression Steroid (XM_011326020.1, biosynthesis)	Designed in this study
FGSG_05740-R	CCGATGTTGTGAGCGAGGTA		
FGSG_04092-F	AATCCTGCGGAAAGTCAAGAG	Quantitative real-time PCR of *FGSG_04092* expression (XM_011323246.1, Steroid biosynthesis)	
FGSG_04092-R	CGGTAACTGTAGGTGAAGCCAT		
FGSG_05011-F	TCCCAGCAACATTCTCCTCA	Quantitative real-time PCR of *FGSG_05011* expression (XM_011325190.1, Peroxisome)	
FGSG_05011-R	CTCACAGCCAACAATCCACC		
FGSG_06724-F	AAGAAGAGAGTCGCCATTGC	Quantitative real-time PCR of *FGSG_06724* expression (XM_011328059.1, Ribosome)	
FGSG_06724-R	CCTTTTTTGCTTTCGCTGTC		
FGSG_04903-F	AACCCCAGATGAACCAGGATA	Quantitative real-time PCR of *FGSG_04903* expression (XM_011325070.1, Amino sugar and nucleotide sugar metabolism)	
FGSG_04903-R	GGGAGTGATGTGAAGGAGAGTG		
FGSG_12603-F	GTCCTCCTTCAAACATCACTGC	Quantitative real-time PCR of *FGSG_12603* expression (XM_011324891.1, Amino sugar and nucleotide sugar metabolism)	
FGSG_12603-R	TCCTTATCACCACACCATCCC		
FGSG_10453-F	CACCAGTTCTTTACCCATCCG	Quantitative real-time PCR of *FGSG_10453* expression (XM_011321130.1, Nitrogen metabolism)	
FGSG_10453-R	TGCTGTCCATACACGCTACCA		
FGSG_07803-F	CGCTATTTGCCCTTGGAACT	Quantitative real-time PCR of *FGSG_07803* expression (XM_011329321.1, Glycolysis / Gluconeogenesis)	
FGSG_07803-R	ACGGAGGAAACACTTGCTGG		
FgActin-F	AACATTGTCATGTCTGGTGGTACC	Internal parameters of quantitative real-time PCR (XP_011327086.1)	
FgActin-R	CACTTGCGGTGAACGATTGA		
